# Retraction Note: The P-selectin and PSGL-1 axis accelerates atherosclerosis via activation of dendritic cells by the TLR4 signaling pathway

**DOI:** 10.1038/s41419-026-08455-0

**Published:** 2026-02-05

**Authors:** Zhishuai Ye, Lei Zhong, Shengnan Zhu, Yinuo Wang, Jie Zheng, Shujing Wang, Jianing Zhang, Rongchong Huang

**Affiliations:** 1 Cardiac Center/Division of Cardiovascular Diseases, Beijing Friendship Hospital, Capital Medical University, 100050 Beijing, China; 2 Department of Cardiology, The First Affiliated Hospital of Dalian Medical University, 116011 Dalian, China; 3 Department of Biochemistry and Molecular Biology, Dalian Medical University, 116044 Dalian, China; 4 College of Life Sciences and Pharmacy, Dalian University of Technology, 116027 Dalian, China

Retraction Note to: *Cell Death & Disease* 10.1038/s41419-019-1736-5, published online 01 July 2019

The Editor-in-Chief has retracted this article after irregularities were noted within several figures of this article, specifically:

- Overlap has been noted between the blots in Figure 5
- Image irregularities have been noted in Figure S1C
- Figure. 2E overlaps with Fig. 2A and B from a previously published article [1]

The Editor-in-Chief has therefore lost confidence in the reliabiltiy of the work presented in this article.

The authors have not responded to any correspondence from the publisher about this retraction.
